# The Homœopathic Treatment of Constipation

**Published:** 1883-03

**Authors:** 


### BOOK NOTICES.

The Homœopathic Treatment of Constipation. By H. Bernard, M. D.; translated by T. M. Strong, M. D. Chatterton Publishing Company, Chicago and New York. 1882.

This book is a small volume of 192 pp., constructed after the model of Bell's celebrated monograph on diarrhœa. Under each remedy are given its most important indications and some clinical cases illustrating its action.

At the end of the volume is an excellent little repertory, also upon the model of Dr. Bell's, though not so full as that masterly production.

The author recommends the use of mineral waters, giving the pathogenesis of two or three of them. This recommendation is very likely to be abused by the average practitioner, who will most likely prescribe them *ad libitum*, thus getting the ordinary purgative effect, as when the usual cathartics of the old school are given.

It is to be regretted that alternation of remedies should be recommended. While amelioration may be produced by alternation, yet it is not likely to make a decisive cure. For illustration see this journal, vol. II, p. 13, for case related by Dr. Dunham.

With the exception of this defect the little book under consideration is an eminently practical work that will often assist the physician in the selection of the most similar remedy. W. M. J.

